# Effects of branded health messages on e-cigarette attitudes, intentions, and behaviors: a longitudinal study among youth and young adults

**DOI:** 10.1186/s12889-021-11092-1

**Published:** 2021-06-15

**Authors:** Jessica Rath, Shreya Tulsiani, W. Douglas Evans, Shiyang Liu, Donna Vallone, Elizabeth C. Hair

**Affiliations:** 1 Truth Initiative Schroeder Institute®, Washington, DC USA; 2 Department of Health, Behavior and Society, Johns Hopkins Bloomberg School of Public Health, Baltimore, MD USA; 3 Department of Behavioral and Community Health, University of Maryland School of Public Health, College Park, MD USA; 4 Department of Prevention and Community Health, Milken Institute School of Public Health, The George Washington University, Washington, DC USA; 5 School of Global Public Health, New York University, New York, NY USA

**Keywords:** Brand equity, Health communication, Health campaign, Social marketing, E-cigarette, Vape, Health branding, Social brand

## Abstract

**Background:**

Launched in 2000, the truth campaign was one of the first health-related campaigns to embrace the building of a brand to further amplify its message, such as by building brand equity. Brand equity is an asset that represents the audience’s perception of the brand. Previous research supports that strong brand equity is associated with lower tobacco intentions and behaviors; however, brand equity and its change over time have not been studied as it relates to e-cigarettes. This study examines the effects of change in brand equity on e-cigarette attitudes, intentions, and behaviors among youth and young adults.

**Methods:**

The sample (*N* = 6427) is from the Truth Longitudinal Cohort, a nationally representative, longitudinal cohort of youth and young adults, ages 15–24. Variables include brand equity tobacco scale, demographic characteristics, and e-cigarette use status. The outcomes included anti-e-cigarette attitudes, intentions to use e-cigarettes, and use of e-cigarettes. Multiple and logistic regression models determined the relationship between change in brand equity from respondents at Spring 2018 to Spring 2019 and respondent e-cigarette outcomes at Fall 2019. All models controlled for demographic characteristics and cigarette use.

**Results:**

Mean brand equity scores in Spring 2018 were significantly associated with greater anti-e-cigarette attitudes (β = 0.17, 95% CI: 0.15, 0.19), lower odds of intention to use (OR = 0.75, 95% CI: 0.66, 0.85), and lower odds of current use (OR = 0.81, 95% CI: 0.72, 0.92). Change in brand equity from Spring 2018 to Spring 2019 was significantly associated with greater anti-e-cigarette attitudes (β = 0.09, 95% CI: 0.06, 0.11) and lower odds of intention to use (OR = 0.79, 95% CI: 0.67, 0.93), but not associated with current use behaviors.

**Conclusions:**

Strengthening brand equity is an effective strategy for influencing anti-e-cigarette related attitudes and intentions, much like it is for anti-smoking campaigns. More research needs to be done on the relationship between change in brand equity and e-cigarette behavior to better understand how brand equity can be wielded to influence change in e-cigarette use behavior.

## Background

Teen smoking of combustible cigarettes has declined since 2000, but the use of e-cigarettes, or vaping nicotine, has significantly increased in the past decade. The percentage of high school students who have ever tried e-cigarettes has increased from 4.5% in 2011 to 39.9% in 2020, and the percentage of high school students who used e-cigarettes in the past month has increased from 1.5% in 2011 to 19.6% in 2020 [1]. Of high school students who had currently used any tobacco product in 2020 (23.6%), the majority used e-cigarettes. Due to the high use of e-cigarettes among youth and young adults, the health risks of e-cigarette use are being heavily studied. There are early indications that e-cigarettes containing nicotine can harm the immune system, cause inhalation of carcinogenic substances, and negatively affect respiratory health [2–4]. These negative health effects are a major cause for concern among youth and young adults.

E-cigarette companies have effectively promoted their branded products on digital media by leveraging social media influencers within social network communities focused on e-cigarettes [5], widely reaching youth and young adults, the primary users of digital media [6, 7]. These marketing strategies closely mimic those of early cigarette advertising to create a positive social norm for using e-cigarettes. JUUL’s “Vaporized” campaign presented a youth-forward brand image by depicting young models using e-cigarettes among colorful, eye-catching backgrounds and references to pop culture [8, 9]. “Vaporized” launched in 2015 and spread across digital platforms with the help of other pro-social network communities [8, 9].

In 2017, the national truth campaign leveraged their success with decreasing the rates of cigarette use among youth and young adults [10, 11] to combat the emerging e-cigarette trend using marketing efforts that directly counteract e-cigarette industry marketing practices. The truth*-*branded, anti-e-cigarette campaign is focused on changing social norms, which have partly made this behavior so popular. The continuity in brand marketing strategies is solidified in content presentation, arming a new generation of potential and current e-cigarette users with the facts they need to make decisions on product use. In order to reach the youth and young adult audience, the truth-branded counter-marketing messages are largely disseminated on digital media using many of the promotional strategies wielded by the tobacco industry, including social media influencers.

“Brand equity,” measured by a multi-dimensional scale, assesses the perceptions of a brand and the value of its product, service, or message [12, 13]. Years of social marketing research has shown that brand equity mediates positive attitudes toward [14] and performance of a desired behavior [15, 16]. For example, studies specific to truth’s campaigns have shown brand equity to be effective in reducing smoking among youth and young adults by mediating the effects of campaign exposure [17]. Studies indicated that positive brand equity, distinct from the content of the messages, functioned as a protective factor for those who had never smoked and reduced self-reported intentions to smoke and past 30-day smoking among ever and current smokers [18–20].

While substantial evidence exists on the effectiveness of branding for the truth counter-marketing campaign for combustible cigarettes, the influence of branding has not yet been evaluated for its e-cigarette messaging strategies. This study builds upon the longitudinal analysis by Evans et al. (2017) [14] that found higher overall brand equity at baseline predicted less cigarette smoking and more positive anti-tobacco attitudes 1 y later, and that an increase in brand equity over time predicted more positive anti-tobacco attitudes 1 y later. The purpose of this study is to assess the role of anti-tobacco brand equity in influencing e-cigarette attitudes, intentions, and behaviors among youth and young adults over time. A longitudinal sample was utilized to examine change in brand equity over 1 y in relation to e-cigarette attitudes, intentions, and behavior 18 months after baseline.

## Methods

### Study design

Data were collected from The Truth Longitudinal Cohort (TLC), a US nationally representative address-based custom panel which began in April 2014 with a baseline sample of approximately 14,000 respondents at baseline. Respondents received follow up surveys every 6 to 12 months [21]. This study was conducted on data collected over 18 months from The TLC utilizing three consecutive time points: Spring 2018, Spring 2019, and Fall 2019. Recruited participants ages 15-24 were sent email invitations with a link to begin the 30-min survey. All study procedures were reviewed and approved for human subjects research by Advarra Institutional Review Board.

### Sample

Only those respondents who participated in the TLC Spring 2018 and Spring 2019 surveys and self-reported recognition of the truth logo were asked the brand equity items. There is high enough logo recognition (75–93%) at each timepoint that the brand equity items go to nearly the full sample and responses are variable, including a range resulting in high and low equity. Those who responded to the brand equity items in Spring 2018 and Spring 2019 and had data for outcomes at Fall 2019 (*N* = 6427) were included in the analyses. Data from Spring 2018 was used for demographic information while study outcomes were collected from the Fall 2019 data.

### Measures

#### Truth brand equity scale

In 2005, Evans et al. demonstrated that four brand equity constructs formed a higher order brand equity factor that mediated the effects of exposure to the original truth campaign on adolescent smoking outcomes [22]. The full brand equity scale is comprised of 16 items representing the following four constructs: loyalty to the brand, leadership and popularity of the brand, brand personality, and brand awareness as it relates to tobacco. Response choices for all brand equity items were on a 5-point Likert scale, ranging from 1) “strongly disagree,” 2) “disagree,” 3) “neither agree or disagree,” 4) “agree,” to 5) “strongly agree.” See Tables 1 and 2 for the individual items in each scale.

**Table 1:** Sample descriptive statistics (*N* = 6427)

Brand Equity Scale	Individual Brand Equity Items	Spring 2018	Spring 2019
	**How much do you agree or disagree with the following?**	**% agree/strongly agree (SE)**	**% agree/strongly agree (SE)**
**Brand Loyalty**	I’d like to help truth make a difference in my generation	42.4 (0.49)	43.8 (0.50)
	I’d defend truth on social media if someone were putting it down	32.6 (0.47)	29.9 (0.46)
	I’d follow truth on social media	25.5 (0.44)	23.5 (0.42)
**Leadership/Popularity**	truth is for people like me	38.9 (0.49)	40.1 (0.49)
**Brand Personality**	**How much do you agree or disagree with** **the following? Truth is … .**		
	Inspired	72.3 (0.45)	69.9 (0.46)
	Powerful	67.2 (0.47)	65.2 (0.48)
	In control of their own decisions	78.0 (0.41))	78.3 (0.41)
	Independent	74.0 (0.44)	74.3 (0.44)
	Honest	75.8 (0.43)	75.3 (0.43)
	Innovative	62.7 (0.48)	61.6 (0.49)
**Brand Awareness**	**When you think of truth, you think …**?		
	Tobacco companies lie	77.7 (0.42)	76.9 (0.42))
	The tobacco industry tries to get young people to smoke other products like hookah	57.7 (0.49)	60.8 (0.49)
	Tobacco company ads are a joke	56.0 (0.50)	58.7 (0.49)
**Demographics (Spring 2018)**			
**Gender (%)**	Female	59.3	
	Male	40.7	
**Race/Ethnicity (%)**	Non-Hispanic White	64.4	
	Non-Hispanic Black	11.3	
	Hispanic/Latino	15.8	
	Non-Hispanic other	8.5	
**Self-Described Financial Situation (%)**	Live comfortably	37.5	
	Meet needs with a little left	38.9	
	Just meet basic expenses, and	18.4	
	Do not meet basic expenses	5.2	
**Education (%)**	Less than high school	14.1	
	High school graduate	17.5	
	Some college/associates degree	37.5	
	College graduate or more	30.9	
**Parent Education (%)**	Less than high school	3.8	
	High school graduate	11.8	
	Some college/associates degree	23.4	
	College graduate or more	61.0	
**Mean (SD)**	Age	21.2 (2.7)	
	Sensation seeking	3.0 (0.8)

**Table 2:** Sample outcome statistics in Fall 2019 (*N* = 6427)

	Spring 2018	Spring 2019	Fall 2019
Anti-e-cigarette attitudes (Mean (SD))			3.3 (0.6)
Intention to use e-cigarettes (% Definitely Yes/Probably Yes)			15.4
Current use of e-cigarettes (% Yes)	13.9 (0.34)	16.5 (0.37)	14.6 (0.35)

In 2016, the brand equity scale was revised and validated to include the same four constructs but with updated content to reflect the truth FinishIt campaign, which focused on ending combustible cigarette use [23]. Confirmatory factor analysis (CFA) was conducted on each brand equity construct when data collection was completed in Spring 2018 (baseline) and Spring 2019, and then on a full brand equity scale created by combining each brand equity construct [23]. Four factors were confirmed —brand loyalty, leadership/popularity, brand personality and brand awareness, as well as an overall brand equity scale (mean of all four factors). This scale based on combustible cigarettes, consisting of 13 items, was used in the current study of e-cigarette attitudes, intentions, and behaviors.

#### Truth logo recognition

Respondents were shown the truth brand logo and were asked “Do you recognize this logo?” with response options “yes” or “no”. Those that responded “yes” were “truth brand aware” and received the full brand equity scale while those who responded “no” were “not truth brand aware” and were not included in analyses.

#### Anti-E-cigarette attitudes

In previous formative work, participants were asked to endorse a series of e-cigarette related attitudinal items. Items were considered as message targets for the campaign if 1) less than 70% of respondents already endorsed the item and 2) if the item was correlated with the outcome of interest (intention not to use e-cigarettes). Following an exploratory factor analysis to identify relationships among items, 3 message theme constructs making up anti-vape sentiment (AVS) were identified: social acceptability of vaping, harms knowledge of vaping, and vaping un-appeal. The constructs were calculated using the average of the attitudinal items within each:

*Social acceptability of vaping* included the following four items: “Vaping/Using E-Cigarettes including JUUL … 1) is ok to do socially with friends (reverse-coded) 2) is not okay for people my age 3) bothers me when people do it around me 4) is okay to do to relieve stress (reverse coded) (α = 0.67).

*Harms knowledge of vaping* included the following four items: “Vaping/Using E-Cigarettes including JUUL … 1) contain dangerous chemicals 2) are harmful to your health 3) contain flavors that are safe to use in vapes / e-cigarettes (reverse coded) 4) is safe (reverse coded) (α = 0.73).

*Vaping Un-Appeal* included the following three items: “Vaping/Using E-Cigarettes including JUUL … 1) would be fun to try because of the flavors (reverse coded) 2) would be fun to try because they look high-tech (reverse coded) 3) looks silly or childish (α = 0.69).

Response options for all items included 1 = strongly disagree, 2 = disagree, 3 = neither agree/disagree, 4 = agree, and 5 = strongly agree. Mean scale scores (range: 1 to 5) were used as a study outcome variable.

#### Tobacco use

Tobacco use was assessed with measures of ever vape use, ever cigarette use, past 30-day vape use, past 30-day cigarette use, and intention to use e-cigarettes in the future.

##### Intention to use e-cigarettes

Intention to use e-cigarettes in the future was used to classify participants into a use status group and was measured with two items regularly used by Truth Initiative and other national youth tobacco surveys: 1) “Thinking about the future, if one of your best friends offered you a vape (even one or two puffs) in the coming year, would you use it?” with response options definitely not; probably not; probably yes; and definitely yes; and 2) “Do you think you will use a vape (even 1 or 2 puffs) in the next year?” with the same response options. Those who responded “probably” or “definitely” yes to either question were coded as having intention to use e-cigarettes.

##### Use status

Participants were asked whether they had ever used (even 1 or 2 times) any of the following: cigarettes, pod-based vapes, vape pens, tank-based vapes, or some other kind of vape. Products they had ever used were included in a follow-up question to assess current use, asking whether they had used the product in the past 30 days (yes; no). Participants who responded “yes” to any vape product were classified as *current users of e-cigarettes.* Participants who responded “yes” to “cigarettes” were classified as *current cigarette users*.

#### Demographic variables

Demographic items included age, gender (male; female) and race/ethnicity (non-Hispanic White; non-Hispanic Black or African American; Hispanic or Latino; non-Hispanic other). Self-described financial situation (live comfortably; meet needs with a little left; just meet basic expenses; do not meet basic expenses), education (less than high school; high school graduate; some college/associates degree; college graduate or more), and an 8-item sensation seeking scale (mean score) [24] were also included as covariates. The sensation seeking scale is on a five-point agreement scale with the following items: I would like to explore strange places; I would like to take off on a trip with no pre-planned routes or timetables; I like to do frightening things; I like wild parties; I like new and exciting experiences, even if I have to break the rules; I get restless when I spend too much time at home; I prefer friends who are exciting and unpredictable; I would like to try parachute-jumping.

### Statistical analysis

The four brand equity factors plus the full scale were utilized in logistic regression models with fixed effects to evaluate the effects of the brand equity scale in Spring 2018 on anti-e-cigarette attitudes, intention to use e-cigarettes, and past 30-day use of e-cigarettes in Fall 2019. A final set of logistic regressions estimated the effects of change in brand equity between Spring 2018 and Spring 2019 on the same outcomes in Fall 2019. All models controlled for age (continuous), gender, race/ethnicity, educational background, parental education, self-described financial situation, sensation seeking, and use status at baseline. Stata version 15.1 was used in all analyses (*Stata Statistical Software: Release 15.1*).

## Results

Overall, participants’ mean age at baseline was 21 years (SD = 2.7), with the majority being female (59%), non-Hispanic white (64%), reporting “Living comfortably” or “Meet needs with a little left” (77% combined) and having some education at the college level or above (69%). Most participants had a parent with a college education or higher (61%). Among the entire study sample, the mean sensation seeking score was 3.0 (SD = 0.8, range 1–5). A total of *N* = 6427 participated in all three waves of data collection for this study: were aware of the truth brand in Spring 2018, responded to the truth brand items again in Spring 2019, and participated in the Fall 2019 data collection (Tables 1 and 2).

Table 3 presents the results of logistic regression models to examine the relationship between the full brand equity scale and each subscale at time one (Spring 2018) and e-cigarette attitudinal/behavioral outcomes 18 months later (Fall 2019). The brand equity constructs measured at time one were significantly associated with most of the outcomes 18 months later. The overall brand equity scale and all subscales in Spring 2018 (time one) were associated with higher anti-e-cigarette attitudes and lower odds of intention to use e-cigarettes in Fall 2019 (18 months later). Lower odds of the behavior, current use of e-cigarettes, in Fall 2019 were only associated with the full brand equity scale, brand loyalty, and brand awareness of tobacco, with all odds ratios less than 0.9.

**Table 3:** Brand equity in Spring 2018 on anti-e-cigarette attitudes, intentions, and use in Fall 2019

Variables at Spring 2018	Model 1: Anti- e-cigarette attitudes coefficient (95% CI) ***n*** = 6337	Model 2: Intention to use e-cigarettes OR (95% CI) ***n*** = 6337	Model 3: Current use of e-cigarettes OR (95% CI) ***n*** = 6337
Brand equity	0.17*** (0.15, 0.19)	0.75*** (0.66, 0.85)	0.81** (0.72, 0.92)
Brand loyalty	0.12*** (0.10, 0.13)	0.77*** (0.70, 0.84)	0.85*** (0.77, 0.93)
Leadership/popularity	0.07*** (0.06, 0.08)	0.88** (0.81, 0.95)	0.92 (0.85, 1.01)
Brand personality	0.11*** (0.09, 0.12)	0.88* (0.80, 0.98)	0.91 (0.82, 1.01)
Brand awareness (tobacco)	0.12*** (0.11, 0.14)	0.85** (0.76, 0.94)	0.86** (0.77, 0.95)

Note: Means for each variable are reported. Each cell represents a different model. All models used the same demographics as covariates

Next, a change in brand equity from time one to time two on e-cigarette attitudinal and behavioral outcomes 18 months later was examined using logistic regression models (Tables 4-5). Table 4 reports the effects of change in overall brand equity on e-cigarette outcomes, whereas change in each brand equity subscale on the outcomes is seen in Table 5. Effects of covariates were similar in models shown in both tables; covariates are omitted in Table 5. Findings indicate that a one-point change in any brand equity subscale between Spring 2018 and Spring 2019 was significantly associated with 3–9% (β: 0.03–0.09, *p* < 0.001) greater anti-e-cigarette attitudes. In addition, a one-point change in the overall brand equity scale, popularity/leadership, and brand awareness of tobacco was significantly associated with approximately 21% (OR 0.79, CI 0.67–0.93, *p < 0.05*), 13% (OR 0.87, CI 0.76–0.99, *p < 0.05*), and 13% (OR 0.87, CI 0.78, 0.98, *p* < 0.05) lower odds of intention to use e-cigarettes respectively.

**Table 4:** Change in overall brand equity (Spring 2018 to Spring 2019) on anti-e-cigarette outcomes (Fall 2019)

Variables	Model 4: Anti-e-cigarette attitudes Coeff (95% CI)	Model 5: Intention to use e-cigarettes OR (95% CI)	Model 6: Current use of e-cigarettes OR (95% CI)
**Change in brand equity**	0.09*** (0.06, 0.11)	0.79** (0.67, 0.93)	0.89 (0.75, 1.06)
**Age**	−0.01** (− 0.02, − 0.004)	0.96 (0.92, 1.01)	0.93** (0.89, 0.98)
**Gender (male = ref)**	0.12** (0.10, 0.15)	0.86 (0.72, 1.02)	0.83* (0.69, 0.99)
**Race (white = ref)**			
NH Black or AA	−0.06** (− 0.10, − 0.02)	0.68* (0.50, 0.94)	0.98 (0.73, 1.33)
Hispanic or Latino	−0.05* (− 0.09, − 0.01)	1.14 (0.89, 1.45)	1.17 (0.91, 1.50)
NH Other	− 0.06** (− 0.11, − 0.02)	1.40* (1.05, 1.87)	1.26 (0.93, 1.71)
**Education (college graduate or more = ref)**			
Less than high school	−0.15*** (− 0.21, − 0.08)	1.49 (0.99, 2.23)	1.42 (0.94, 2.14)
High school graduate	− 0.07** (− 0.12, − 0.03)	1.18 (0.86, 1.60)	1.22 (0.89, 1.67)
Some college/AA degree	− 0.07*** (− 0.10, − 0.03)	1.51*** (1.18, 1.92)	1.45** (1.13, 1.86)
**Parent Education (college graduate or more = ref)**			
Less than high school	0.02 (− 0.05, 0.10)	0.56 (0.33, 0.95)	0.73 (0.43, 1.23)
High school graduate	−0.03 (− 0.08, 0.01)	1.16 (0.88, 1.54)	1.23 (0.92, 1.64)
Some college/AA degree	−0.04* (− 0.07, − 0.02)	1.00 (0.81, 1.25)	1.12 (0.90, 1.40)
**Financial situation (Meet needs with leftover = ref)**			
Don’t meet basic expense	−0.11*** (− 0.17, − 0.05)	1.61* (1.10, 2.35)	1.56* (1.06, 2.29)
Just meet basic expense	−0.10*** (− 0.14, − 0.06)	1.39** (1.08, 1.77)	1.34* (1.039, 1.72)
Meet needs with a little left	− 0.04** (− 0.07, − 0.01)	0.96 (0.78, 1.18)	0.96 (0.78, 1.19)
**Sensation seeking**	−0.07*** (− 0.08, − 0.05)	1.60*** (1.43, 1.79)	1.53*** (1.36, 1.71)
**Past 30-day cigarette use**	− 0.27*** (− 0.31, − 0.23)	2.82*** (2.28, 3.48)	2.66*** (2.15, 3.30)
**Past 30-day vape use**	−0.41*** (− 0.46, − 0.37)	7.27*** (5.96, 8.86)	8.46*** (6.02, 10.34)

Note: Each column represents a different model

**Table 5:** Change in brand equity subscales (Spring 2018 to Spring 2019) on anti-e-cigarette outcomes (Fall 2019)

Brand Equity Construct	Model 4: Anti-e-cigarette attitudes Coeff (95% CI)	Model 5: Intention to use e-cigarettes OR (95% CI)	Model 6: Current use of e-cigarettes OR (95% CI)
**Change in brand loyalty**	0.05*** (0.03, 0.07)	0.90 (0.80, 1.02)	0.96 (0.85, 1.09)
**Change in leadership/popularity**	0.04*** (0.02, 0.06)	0.87* (0.76, 0.99)	0.94 (0.83, 1.07)
**Change in brand personality**	0.03*** (0.01, 0.04)	0.93 (0.85, 1.02)	0.96 (0.87, 1.05)
**Change in brand awareness (tobacco)**	0.05*** (0.03, 0.07)	0.87* (0.78, 0.98)	0.94 (0.83, 1.06)

Note: Each cell represents a different model. All models used the same demographics as covariates

## Discussion

The truth campaign’s established brand, which was largely focused on preventing combustible cigarette smoking, has been shown to be associated with reduced smoking initiation and use [18, 20]. Previous studies have shown a longitudinal effect of brand equity on smoking outcomes in the context of other anti-tobacco campaigns [18]. This study investigated whether similar effects would be observed in anti-e-cigarette campaigns, building upon truth’s brand health momentum and providing evidence of the influence of the truth tobacco counter-marketing brand equity on e-cigarette use outcomes. Findings indicate that higher brand equity at baseline was significantly associated with greater anti-e-cigarette attitudes and lower odds of intention to use e-cigarettes over time. Significant effects of an increase in brand equity over time on subsequent anti-e-cigarette outcomes were identified. Specifically, a one-point change in the overall brand equity scale, and each of the individual subscales, was associated with an increase in anti-e-cigarette attitudes. Additionally, a one-point increase in the overall scale, and the leadership/popularity and tobacco brand awareness subscales, was associated with a decrease in intention to use e-cigarettes.

These findings are consistent with previous research [18] on the effects of brand equity on outcomes related to combustible use; brand equity continues to be a robust predictor of anti-e-cigarette campaign outcomes. Although the changes in brand equity observed in the study time period were relatively small in total magnitude, these changes were associated with improved anti-e-cigarette outcomes. Brand equity is not only a consistent predictor of improved outcomes over multiple studies, but it is also a highly sensitive measure. While the observed increase in brand equity over time was small, it produced a significant effect on anti-e-cigarette outcomes, indicating that brand equity is a sensitive scale. The truth campaign makes a great effort to remain relevant to our audience in order to maintain or increase brand equity. We did this in part between 2018 and 2019 by pivoting from focusing only on cigarette smoking to including prevention of e-cigarettes in our messaging. Successful efforts to further increase brand equity in truth may produce even greater prevention effects. This hypothesis should be explored in future research.

Future research needs to focus on brand equity scales specific to anti-e-cigarette outcomes, as the current study used the existing tobacco-focused brand equity scale. They should also examine these effects in the context of other anti-e-cigarette campaigns in addition to truth. Additionally, future studies should examine how and to what extent demographic and behavioral factors vary among individuals who have higher and lower levels of brand equity. Moreover, studies should examine the trajectories of brand equity increases over time to help elucidate the mechanisms by which the scale may indicate anti-e-cigarette behavior over time.

There were some limitations to the current study. First, we did not analyze data or compare the current data on tobacco brand awareness to an e-cigarette specific brand awareness subscale. E-cigarette use is a rapidly increasing phenomenon, especially among adolescents and young adults. It is also highly popular and perceptions of risk and personal harm from e-cigarettes differ from combustible use. Also, this study did not include the full address-based random sample, which could affect the results. However, we did not observe any evidence of bias in outcomes in the sub-sample used in this analysis. Lastly, this study is limited in that it cannot attribute causation. While we observed an association between brand equity and anti-e-cigarette attitudes, we cannot rule out reverse causation (i.e., prior attitudes leading to variation in brand equity).

## Conclusions

Brand equity is a robust and highly sensitive construct for assessing how branded anti-e-cigarette messages can shift population-level attitudes, beliefs, and intentions. This research extends previous studies on anti-combustible branding and provides evidence that this counter-marketing strategy is effective in preventing e-cigarette use as well. Brands are powerful tools for public health interventions, especially in tobacco and nicotine control. National, state, and local public health education efforts can benefit by the use of branding, particularly for those aimed at youth and young adults.

## Data Availability

The dataset used in the current study is available by request with a data use agreement. Please make requests with Dr. Jessica Rath at jrath@truthinitiative.org.

## Abbreviations

TLC: Truth Longitudinal Cohort
CFA: Confirmatory factor analysis
AVS: Anti-vape sentiment

## References

[1] Centers for disease control and prevention (CDC). National Youth Tobacco Survey (NYTS). 2020.

[2] Lei W, Lerner C, Sundar IK, Rahman I (2017). Myofibroblast differentiation and its functional properties are inhibited by nicotine and e-cigarette via mitochondrial OXPHOS complex III. Sci Rep.

[3] McConnell R, Barrington-Trimis JL, Wang K, Urman R, Hong H, Unger J, Samet J, Leventhal A, Berhane K (2017). Electronic cigarette use and respiratory symptoms in adolescents. Am J Respir Crit Care Med.

[4] Pankow JF, Kim K, McWhirter KJ, Luo W, Escobedo JO, Strongin RM (2017). Benzene formation in electronic cigarettes. PLoS One.

[5] Czaplicki L, Kostygina G, Kim Y, Perks SN, Szczypka G, Emery SL, Vallone D, Hair EC (2020). Characterising JUUL-related posts on Instagram. Tob Control.

[6] Center PR. Teens, social media & technology 2018. 2018.

[7] Center PR. Share of U.S. adults using social media, including Facebook, is mostly unchanged since 2018. 2019.

[8] Huang J, Duan Z, Kwok J, Binns S, Vera LE, Kim Y, Szczypka G, Emery SL (2019). Vaping versus JUULing: how the extraordinary growth and marketing of JUUL transformed the US retail e-cigarette market. Tob Control.

[9] Jackler RK, Ramamurthi D (2019). Nicotine arms race: JUUL and the high-nicotine product market. Tob Control.

[10] Farrelly MC, Davis KC, Haviland ML, Messeri P, Healton CG (2005). Evidence of a dose-response relationship between “truth” antismoking ads and youth smoking prevalence. Am J Public Health.

[11] Farrelly MC, Nonnemaker J, Davis KC, Hussin A (2009). The influence of the national truth® campaign on smoking initiation. Am J Prev Med.

[12] Aaker DA. Managing brand equity: the free press; 1991.

[13] Lefebvre R. Social marketing and social change: strategies and tools for improving health, well-being, and the environment: John Wiley & Sons; 2013.

[14] Evans WD, Andrade EL, Barrett ND, Cleary SD, Snider J, Edberg M (2018). The mediating effect of Adelante brand equity on Latino immigrant positive youth development outcomes. J Health Commun.

[15] Evans WD, Blitstein J, Hersey JC, Renaud J, Yaroch AL (2008). Systematic review of public health branding. J Health Commun.

[16] Price SM, Potter LD, Das B, Wang Y-CL, Huhman M (2009). Exploring the influence of the VERB™ brand using a brand equity framework. Soc Mar Q.

[17] Allen J, Vallone D, Vargyas E, Healton CG. The truth campaign: using counter marketing to reduce youth smoking. The new world of health promotion, new program development, implementation and evaluation: Jones & Bartlett Publishers; 2009. p. 195–215.

[18] Evans WD, Rath JM, Hair EC, Snider JW, Pitzer L, Greenberg M (2017). Effects of the truth FinishIt brand on tobacco outcomes. Prev Med Rep.

[19] Hair EC, Holtgrave DR, Romberg AR, Bennett M, Rath JM, Diaz MC, Vallone DM (2019). Cost-effectiveness of using mass media to prevent tobacco use among youth and young adults: the FinishIt campaign. Int J Environ Res Public Health.

[20] Vallone D, Greenberg M, Xiao H, Bennett M, Cantrell J, Rath J, Hair E (2017). The effect of branding to promote healthy behavior: reducing tobacco use among youth and young adults. Int J Environ Res Public Health.

[21] Cantrell J, Hair EC, Smith A, Bennett M, Rath JM, Thomas RK, Fahimi M, Dennis JM, Vallone D (2018). Recruiting and retaining youth and young adults: challenges and opportunities in survey research for tobacco control. Tob Control.

[22] Evans WD, Price S, Blahut S (2005). Evaluating the truth® brand. J Health Commun.

[23] Evans WD, Rath J, Pitzer L, Hair EC, Snider J, Cantrell J, Vallone D (2016). Design and feasibility testing of the truth FinishIt tobacco countermarketing brand equity scale. J Health Commun.

[24] Hoyle RH, Stephenson MT, Palmgreen P, Lorch EP, Donohew RL (2002). Reliability and validity of a brief measure of sensation seeking. Pers Individ Dif.

